# Supplementary material for: Clinical Efficacy of Ruxolitinib in Patients with Myelofibrosis: A Nationwide Population-Based Study in Korea
Source: J Clin Med. 2021 Oct 18;10(20):4774. doi: 10.3390/jcm10204774 (PMC8540308; doi:10.3390/jcm10204774)
Supplement: Supplementary file 1 [file jcm-10-04774-s001.zip › jcm-1400406-supplementary.pdf]

Table S1. Stratified Cox regression analysis for occurrence of leukemia.

	Unmatched						Propensity Score Matched					
	n	No. of Events	HR	95% CI	*p*	*p* for Interaction	*n*	No. of Events	HR	95% CI	*p*	*p* for Interaction
Overall												
No ruxolitinib	507	61	0.83	0.55–1.26	0.384		224	25	0.78	0.48–1.28	0.331	
Ruxolitinib	224	36	1.00				224	36	1.00			
						0.444						0.231
Primary MF												
No ruxolitinib	291	38	0.70	0.41–1.19	0.182		113	11	0.59	0.29–1.15	0.121	
Ruxolitinib	100	21	1.00				100	21	1.00			
Secondary MF												
No ruxolitinib	216	23	0.97	0.50–1.85	0.916		111	14	1.08	0.52–2.22	0.845	
Ruxolitinib	124	15	1.00				124	15	1.00			

CI, confidence interval; HR, hazard ratio; MF, myelofibrosis; OS, overall survival.

Table S2. Stratified Cox regression analysis for thrombotic complications.

	Unmatched						Propensity Score Matched					
	n	No. of Events	HR	95% CI	*p*	*p* for Interaction	*n*	No. of Events	HR	95% CI	*p*	*p* for Interaction
Overall												
No ruxolitinib	507	35	0.95	0.54–1.69	0.866		224	15	0.94	0.48–1.85	0.867	
Ruxolitinib	224	18	1.00				224	18	1.00			
						0.500						0.499
Primary MF												
No ruxolitinib	291	17	0.79	0.35–1.76	0.564		113	9	1.18	0.47–2.94	0.721	
Ruxolitinib	100	9	1.00				100	9	1.00			
Secondary MF												
No ruxolitinib	216	18	1.17	0.52–2.63	0.706		111	6	0.72	0.25–2.08	0.551	
Ruxolitinib	124	9	1.00				124	9	1.00			

CI, confidence interval; HR, hazard ratio; MF, myelofibrosis; OS, overall survival.
